# Cholestatic liver injury due to leukemic infiltration in *HOX11*-positive acute monocytic leukemia: a case report

**DOI:** 10.3389/fonc.2025.1620271

**Published:** 2025-08-06

**Authors:** Huiping Xu, Qunqing She, Linjun Xie

**Affiliations:** ^1^ The First Hospital of Putian City, Putian, China; ^2^ The School of Clinical Medicine, Fujian Medical University, Fuzhou, China

**Keywords:** acute monocytic leukemia, HOX11, leukemic hepatic infiltration, cholestatic liver injury, venetoclax-azacitidine

## Abstract

We report the case of a 78-year-old male who was diagnosed with *HOX11*-positive acute monocytic leukemia (AML-M5), complicated by leukemic hepatic infiltration and cholestatic liver injury. Initial management included hydroxyurea and liver-protective therapies; however, liver dysfunction progressed despite treatment. With the patient’s liver function deteriorating, chemotherapy with venetoclax and azacitidine was initiated under close monitoring, along with intensive supportive care including methylprednisolone. This regimen choice was based on a careful assessment of the hepatotoxicity profiles of these drugs in conjunction with the patient’s hepatic function. As the leukemic burden decreased, liver function gradually improved, and the patient achieved hematologic recovery sufficient for discharge. This case highlights the challenges of treating elderly AML-M5 patients with hepatic infiltration and emphasizes the importance of early recognition and individualized treatment strategies and the potential benefits of dose-adjusted induction therapy tailored according to the hepatotoxicity profiles of the drugs and the patient’s hepatic function.

## 1 Introduction

Acute monocytic leukemia (AML-M5) is a highly aggressive subtype of acute myeloid leukemia, characterized by a marked proliferation of monoblasts and promonocytes. Extramedullary infiltration is a frequent clinical feature, most commonly affecting the gingiva, skin, and central nervous system (1). *HOX11*, also known as *TLX1*, is primarily associated with acute lymphoblastic leukemia (ALL), and its presence in AML is relatively rare. Its expression correlates with a relatively favorable prognosis (2, 3). However, the clinical significance of *HOX11* in AML remains underexplored.

Leukemic hepatic infiltration (LHI) typically presents with hepatomegaly, elevated transaminase levels, and features of cholestatic liver injury (4–18). Such presentations can closely mimic other conditions, including viral hepatitis, tumor lysis syndrome (TLS), drug-induced liver injury, or biliary obstruction, often complicating timely diagnosis. Although liver biopsy remains the gold standard for confirming LHI, this procedure is frequently contraindicated due to the profound thrombocytopenia that often accompanies leukemia. In this context, noninvasive approaches, including viral serologies, immunological assays, and imaging modalities such as contrast-enhanced computed tomography (CT) or magnetic resonance imaging (MRI), are essential for exclusion-based diagnosis and for assessing the extent of hepatic infiltration (19, 20).

The management of patients with significant hepatic injury prior to chemotherapy remains particularly challenging, especially in elderly patients, as no standardized treatment strategies have been established. Clinical decision-making must balance the urgency of disease control against the risk of exacerbating liver injury, favoring regimens with minimal hepatotoxicity and necessitating close monitoring of hepatic parameters (18). Recent advances, such as the combination of venetoclax and azacitidine, have substantially improved outcomes in elderly or frail patients with AML (21, 22). However, using azacitidine is generally discouraged in cases of moderate to severe hepatic impairment due to its potential for hepatotoxicity, and venetoclax dosing requires careful adjustment based on liver function (23, 24). Crucially, data regarding the use of this regimen in elderly AML-M5 patients with severe hepatic injury are currently limited, highlighting the need for individualized treatment approaches within this population.

Here, we report a rare case of an elderly patient with *HOX11*-positive AML-M5 who presented with severe cholestatic liver injury prior to the initiation of chemotherapy. Despite initial worsening of liver injury following treatment initiation, continued cytoreductive therapy led to a significant reduction in leukemic burden, normalization of peripheral blood counts, and progressive improvement in liver function. This case highlights the importance of recognizing LHI as a potential cause of liver injury in AML and suggests that effective cytoreductive therapy may offer reversibility even in cases complicated by profound hepatic impairment. Informed consent was obtained from the patient’s family.

## 2 Case description

A 78-year-old male was admitted to our hospital with a 20-day history of persistent cough and generalized fatigue. The cough was productive, with white, mucoid sputum, and the fatigue was exacerbated by physical activity, alleviated by rest. The patient denied experiencing chills, fever, epistaxis, gingival bleeding, melena, bone pain, dark urine, or jaundice. Despite receiving cefixime for a suspected respiratory tract infection in an outpatient setting, his symptoms showed minimal improvement. Upon admission, his vital signs were stable, with a temperature of 36.3°C, pulse rate of 78 beats per minute, respiratory rate of 19 breaths per minute, and blood pressure of 125/78 mmHg. He appeared pale and fatigued but was conscious and alert. Chest examination revealed coarse breath sounds and a few moist rales. Cardiovascular examination showed a regular rhythm without murmurs. Abdominal examination revealed a soft abdomen with no tenderness or rebound tenderness, and neither the liver nor the spleen was palpable.

**Figure 1:**
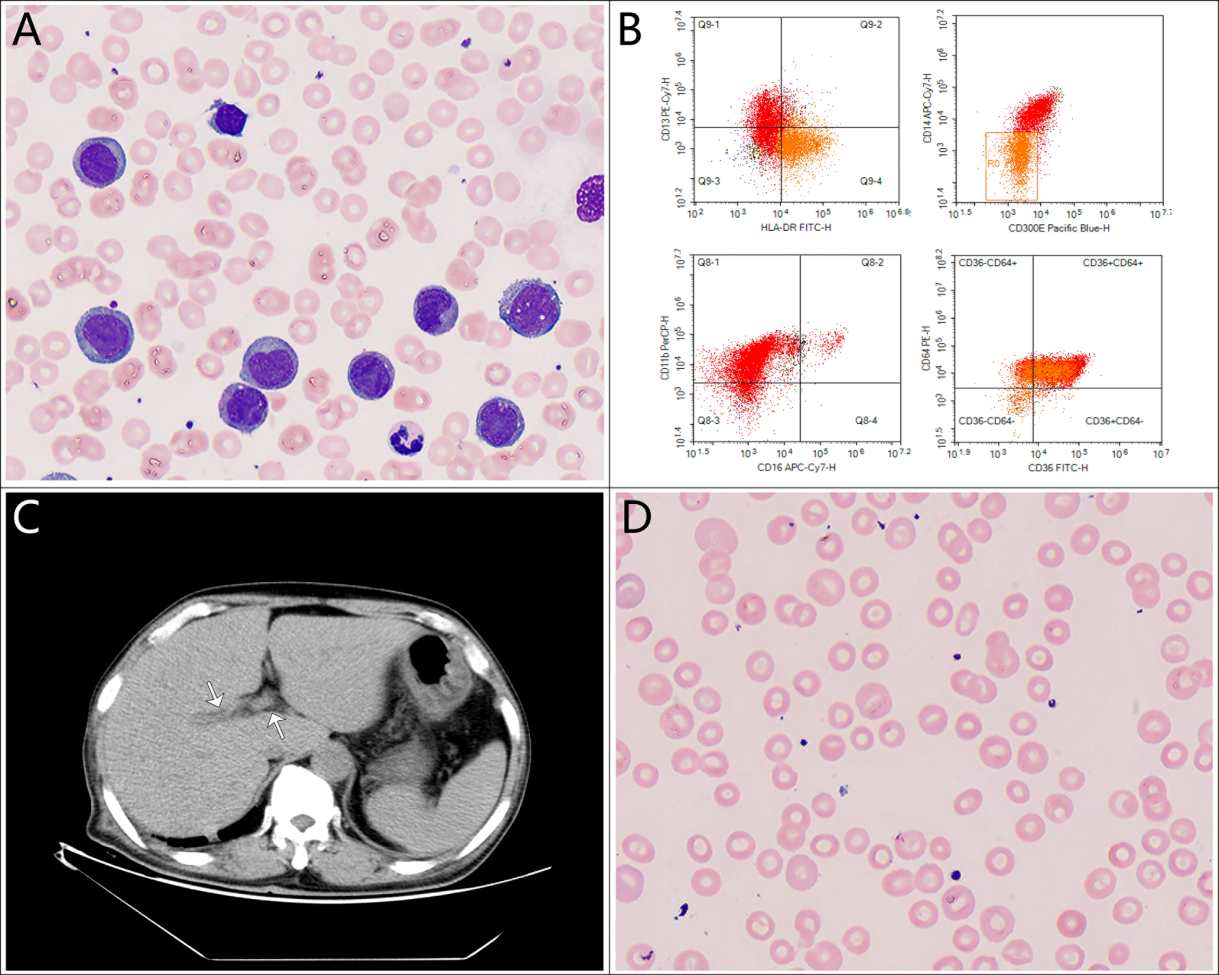
Key diagnostic findings in this case. **(A)** Peripheral blood smear showing a significant number of immature monocytes; **(B)** Bone marrow flow cytometry confirming HLA-DR^+^CD14^-^CD300e^-^ immature monocytes; **(C)** Abdominal CT scan showing edema of Glisson’s capsule; **(D)** Peripheral blood smear with target cells.

Laboratory tests on admission revealed an elevated white blood cell count (WBC) of 86.01 × 10^9^/L, with 35% blasts, a hemoglobin level (Hb) of 108 g/L, and a platelet count (PLT) of 163 × 10^9^/L. A peripheral blood smear showed a significant number of immature monocytes (see **Figure 1A**), and bone marrow aspiration revealed 92.5% blasts. Bone marrow flow cytometry confirmed 96.33% blasts/immature monocytes and 42.31% HLA-DR^+^CD14^-^CD300e^-^ cells (see **Figure 1B**). The leukemia fusion gene panel, performed using quantitative real-time PCR (qRT-PCR), revealed positivity for the HOX11 gene, while all other fusion genes tested were negative. Cytogenetic analysis revealed a karyotype: 45,X,-Y. C-reactive protein (CRP) was mildly elevated at 26.18 mg/L. Liver function tests showed total protein (TP) at 56.2 g/L and albumin (Alb) at 31.7 g/L, with elevated total bilirubin (T-Bil) at 35.6 µmol/L, direct bilirubin (D-Bil) at 24.9 µmol/L, alanine aminotransferase (ALT) at 110 U/L, aspartate aminotransferase (AST) at 123 U/L, gamma-glutamyl transferase (GGT) at 325 U/L, alkaline phosphatase (ALP) at 265 U/L, lactate dehydrogenase (LDH) was markedly elevated at 1284 U/L, prothrombin time (PT) at 16.4 seconds, activated partial thromboplastin time (APTT) at 42.8 seconds, and prothrombin activity (PTA) at 57.3%. These findings suggest significant liver dysfunction. Renal function was slightly impaired, with a serum creatinine level of 135 µmol/L. Autoimmune hepatitis markers, including antinuclear antibodies (ANA), smooth muscle antibodies (SMA), liver/kidney microsomal antibodies (LKM), and anti-mitochondrial antibodies (AMA), were all negative. Additionally, tests for hepatitis viruses (HBsAg, HCV antibodies, HEV antibodies, HAV IgM antibodies), Epstein–Barr virus (EBV), and cytomegalovirus (CMV) were negative. A chest CT scan revealed bilateral pulmonary infiltrates, suggesting an underlying infection. Abdominal CT suggested exudative changes in the gallbladder, with low-density lesions around hepatic vasculature, which was considered consistent with perivascular edema of Glisson’s sheath (see **Figure 1C**, arrows), no biliary duct dilation or stones were observed, bilateral renal cysts were noted.

The patient was initially diagnosed with *HOX11*-positive AML-M5 and classified as adverse risk, with secondary diagnoses including pulmonary infection and cholestatic liver injury. Based on the clinical presentation and laboratory findings, broad-spectrum antibiotics were initiated, along with ursodeoxycholic acid and ademetionine 1,4-butanesulfonate to manage liver function. Due to the patient’s age and high tumor burden, hydroxyurea was administered initially. Despite these treatments, the patient’s WBC, T-Bil, and D-Bil continued to rise. On admission, the patient already exhibited liver dysfunction, which did not meet the criteria for TLS. Further investigations excluded viral hepatitis, autoimmune hepatitis, choledocholithiasis, and cholangitis, leading to a clinical diagnosis of LHI. A liver biopsy was recommended, but the patient’s family declined due to the patient’s advanced age and frailty.

**Figure 2:**
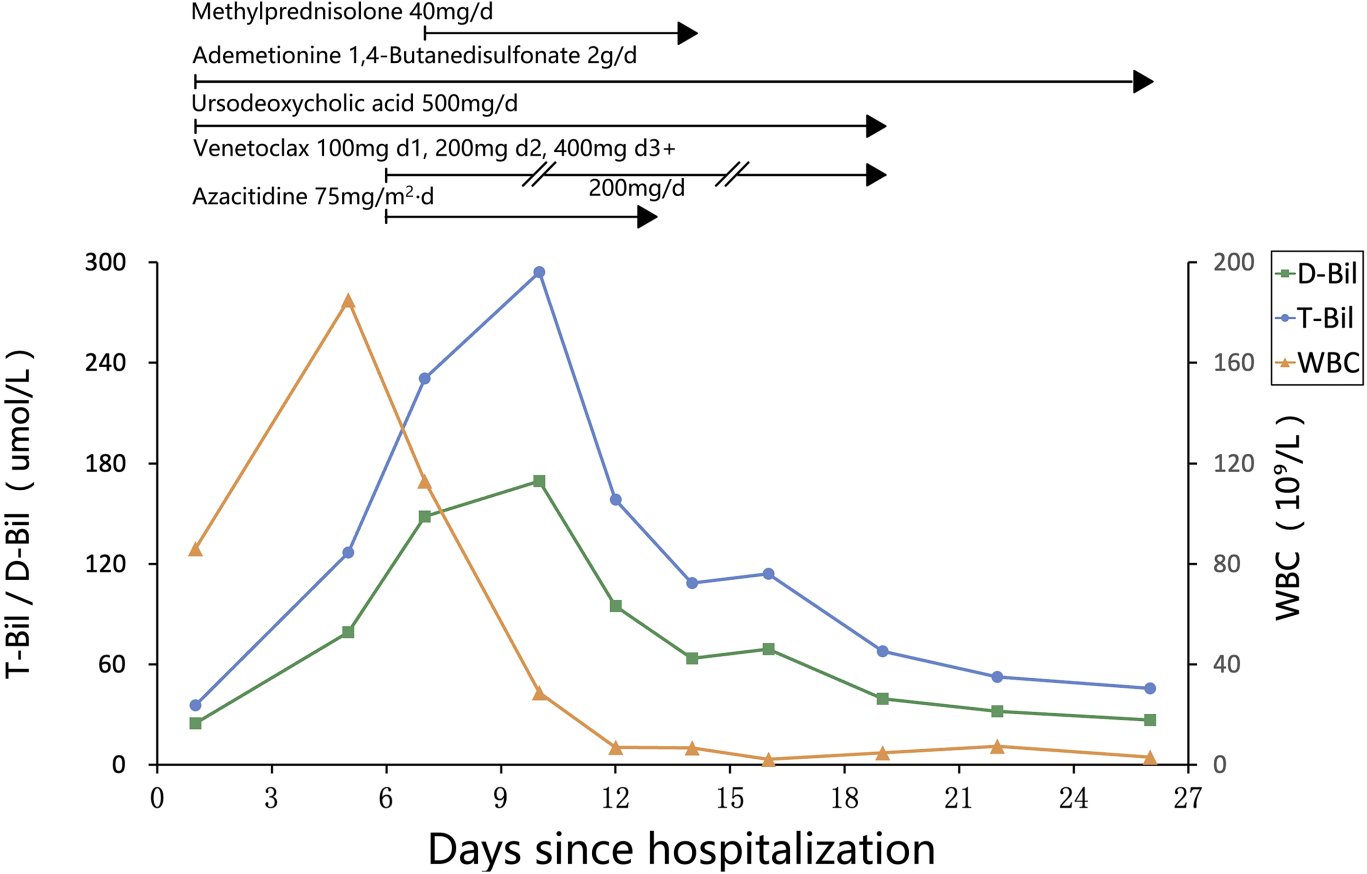
Changes in medication regimen and blood parameters during hospitalization.

Chemotherapy with venetoclax and azacitidine was initiated on day 6 of hospitalization. WBC started to decrease, but bilirubin levels rose further, and target cells were noted on the peripheral blood smear (see **Figure 1D**). The venetoclax dose was temporarily reduced. In addition to continuing ursodeoxycholic acid, ademetionine 1,4-butanesulfonate, methylprednisolone was also added. As the patient’s WBC continued to decrease, bilirubin levels also showed improvement. However, on day 19, the patient displayed signs of pessimism and refused oral medications, opting only for intravenous nutrition. By day 26, the patient’s condition had significantly improved, with PLT increasing from a nadir of 1 to 182 × 10^9^/L, and he was subsequently discharged from the hospital. The detailed medication regimen and changes in blood parameters are shown in **Figure 2** and **Table 1**.

**Table 1:** Changes in blood parameters during hospitalization.

Day	WBC	Hb	PLT	TP	ALB	T-Bil	D-Bil	ALT	AST	GGT	ALP	LDH	PT	APTT	PTA
	10^9^/L	g/L	10^9^/L	g/L		µmol/L		U/L					s		%
1	86.01	108	163	56.2	31.7	35.6	24.9	110	123	325	265	1284	16.4	42.8	57.3
5	185.03	93	144	51.6	30.5	126.8	79.1	76	78	242	305	1401	17.7	51.2	51
7	113.05	103	63	51.8	29.4	230.8	148.3	295	174	265	222	814	16.2	55.9	58.4
10	28.79	83	22	–	–	294.1	169.3	163	44	213	214	720	12.4	37.6	87.1
12	6.98	82	11	55.6	33.1	158.4	94.8	124	52	190	200	523	12.3	43.2	88.3
14	6.83	73	12	52.7	32.3	108.6	63.5	93	30	139	189	366	13.1	36	79.1
16	2.35	54	20	48.4	29.0	114	69.1	57	20	96	170	310	–	–	–
19	4.79	79	1	46.9	27.3	67.8	39.6	34	15	96	171	242	13.4	48.9	76
22	7.48	71	32	52.3	26.7	52.5	32	33	19	15	195	221	13.2	35.3	78
26	3.2	72	182	–	–	45.8	26.7	30	17	16	172	218	13	35.2	79

After discharge, the patient’s subjective symptoms continued to improve. He gradually resumed oral intake and continued venetoclax therapy. Despite multiple telephone follow-ups recommending readmission for further treatment, the patient declined. One month later, laboratory re-evaluation showed WBC 7.08 × 10^9^/L, Hb 77 g/L, and PLT 238 × 10^9^/L. Biochemical tests revealed T-Bil 6.7 µmol/L, D-Bil 3.3 µmol/L, ALT 7 U/L, AST 13 U/L, GGT 15 U/L, ALP 90 U/L, serum creatinine 111 µmol/L, and LDH 193 U/L. Although laboratory parameters showed marked improvement, the patient continued to refuse hospitalization and repeat bone marrow aspiration; therefore, measurable residual disease (MRD) assessment and systematic evaluation of treatment response could not be performed. The patient was subsequently lost to follow-up.

This case highlights the challenges of managing AML-M5 in elderly patients, especially when complicated by LHI and cholestatic liver injury. It emphasizes the critical role of early detection and multidisciplinary care in addressing liver dysfunction during leukemia treatment. The case also reflects the need to carefully balance effective cytoreduction with the risk of organ toxicity, showing that with vigilant monitoring and strong supportive measures, aggressive antitumor therapy can still be safely pursued.

## 3 Discussion

The prevalence and clinical presentation of LHI vary depending on the specific leukemia subtype. In ALL, hepatic infiltration is relatively common. Studies have shown that more than 30% of pediatric patients with newly diagnosed ALL present with asymptomatic hepatomegaly and elevated serum AST and ALT levels, although hyperbilirubinemia is observed in only approximately 3.4% of cases (10). Notably, acute liver failure as the initial presentation of ALL is exceedingly rare (5–8). In contrast, hepatomegaly is less frequently observed in patients with AML. Nevertheless, autopsy studies have demonstrated LHI in up to 75% of AML cases, particularly among those with the AML-M5 subtype (25). Overall, ALL is typically associated with diffuse hepatomegaly and mild elevations in liver enzymes, whereas AML, particularly the M5 subtype, is more frequently associated with cholestasis; however, severe hepatic injury remains rare in both ALL and AML (26). Given these differences, careful assessment of the leukemia subtype is essential when evaluating hepatic dysfunction in patients with acute leukemia, as the underlying pathology may impact both clinical presentation and therapeutic decision-making.

LHI is a multifaceted process involving both cellular and microenvironmental factors. The expression of liver-specific chemokine receptors, such as CCR1, CCR2, and CCR5, on leukemic cells facilitates their homing to the liver, where they acquire leukemia stem cell (LSC)-like properties, including enhanced self-renewal and proliferative capacity (27). Moreover, the hepatic microenvironment promotes leukemic cell survival by inducing metabolic adaptations, enhancing proliferation via polyunsaturated fatty acid pathways, stabilizing anti-apoptotic proteins, and degrading chemotherapy drugs (28). These pathological changes not only support leukemic cell persistence but also contribute to hepatic dysfunction. Specifically, LHI can induce sinusoidal obstruction and tissue ischemia, leading to elevated transaminase levels and, in severe cases, progressing to hepatic necrosis and acute liver failure (9–12). Furthermore, granulocytic sarcoma may cause obstructive jaundice by compressing or infiltrating the bile ducts (12–15). Both mechanisms impair hepatic clearance, resulting in the accumulation of lactate and bilirubin, which further exacerbates liver injury and creates a vicious cycle of metabolic dysfunction and hepatocellular damage (29, 30).

The patient was diagnosed with AML-M5 and exhibited a continuous increase in WBC, T-Bil, and D-Bil after admission. Laboratory findings revealed that GGT and ALP levels were elevated more significantly than ALT and AST, consistent with a pattern of obstructive jaundice, similar to findings reported in previous cases (11–18). Cefixime and hydroxyurea, both primarily renally excreted with minimal hepatic metabolism and low risk of cholestatic liver injury, were used early in the course and were considered unlikely contributors based on their pharmacokinetics and the timing of liver dysfunction. CT imaging demonstrated perivascular edema of Glisson’s sheath without evidence of hepatomegaly or biliary duct dilatation. Immunologic and virologic workups excluded autoimmune hepatitis and viral hepatitis, and there was no history of exposure to suspicious hepatotoxic drugs, making drug-induced liver injury unlikely. Although liver biopsy could not be performed due to family refusal, the combination of clinical manifestations, laboratory abnormalities, and the known high extramedullary aggressiveness of AML-M5 strongly supported the diagnosis of LHI. Of particular interest, the patient was positive for *HOX11*, a genetic finding not previously reported in AML cases with LHI, the significance of which remains to be elucidated.

*HOX11* is a homeobox gene that encodes a transcription factor critical for embryonic development and T-cell differentiation (31, 32). In T-ALL, overexpression of *HOX11* can lead to the immortalization of hematopoietic progenitor cells with both primitive and definitive hematopoietic potential (33, 34). However, its specific role in AML and LHI remains unclear. Spinelli et al. (11) reported a case of cholestasis associated with *CBFB–MYH11*-positive AML, in which liver infiltration by leukemic cells was observed. Similarly, in Maharaj’s review, four additional cases of LHI were also *CBFB–MYH11*-positive (18). Given that *CBFB–MYH11*-positive AML often displays monoblastic/monocytic features and a tendency toward abdominal myeloid sarcoma, these cases highlight the potential of *CBFB–MYH11* to promote hepatic involvement (11). Whether *HOX11*, like *CBFB–MYH11*, contributes to leukemic cell migration and hepatic infiltration warrants further investigation.

In patients with AML-LHI, delaying induction therapy carries the risk of concurrent progression of both leukemia and hepatic dysfunction. Currently, no standardized guidelines exist for this rare presentation. Limited case reports suggest that prognosis is particularly poor in infants and elderly patients (4, 12, 15, 17). Adult patients may be treated with reduced- or full-dose cytarabine and anthracycline-based regimens, depending on liver function status (11, 13, 14, 16, 18). In cases of severe hepatic impairment, hydroxyurea has been used as a cytoreductive bridge to induction chemotherapy (16). One report described worsening jaundice during chemotherapy, which improved after endoscopic biliary decompression, suggesting that interventional management of cholestasis may improve outcomes (14). Additionally, early administration of corticosteroids appears to be more effective than conventional hepatoprotective agents in mitigating liver injury (5, 10).

We hypothesize that poor prognosis in elderly patients may be partly attributed to treatment delays or omission of therapy. Achieving hematologic remission is critical, as it often leads to resolution of hepatic infiltration. However, in elderly patients with poor performance status and liver dysfunction, therapeutic options are extremely limited. The emergence of targeted agents such as FLT3 inhibitors, IDH1/2 inhibitors, and BCL-2 inhibitors (e.g., venetoclax) provides promising alternatives for patients unfit for intensive chemotherapy (21, 22, 35, 36). To our knowledge, the present case involves the oldest reported AML-LHI patient. Following an unsuccessful attempt at cytoreduction with hydroxyurea, we promptly initiated a dose-adjusted venetoclax-azacitidine regimen with adjunctive methylprednisolone, which led to improvement in both peripheral counts and hepatic function.

Given the rarity of LHI and the absence of standardized management strategies, treatment decisions rely heavily on clinical judgment and individual patient characteristics. The lack of prospective data and reliance on isolated case reports limit the generalizability of current treatment approaches. Future studies are needed to define optimal therapeutic strategies and clarify the role of novel agents in this challenging patient population.

## 4 Conclusion

LHI is a rare but serious complication of AML that presents significant therapeutic challenges, particularly in the elderly. This case underscores the importance of early recognition and timely, individualized treatment. Dose-adjusted induction therapy based on hepatic function and drug toxicity profiles may offer clinical benefit. Further studies are needed to optimize management strategies and clarify the role of novel agents in AML-LHI.

## Data Availability

The data analyzed in this study is subject to the following licenses/restrictions: The datasets presented in this article are not readily available because of ethical/privacy restrictions. Requests to access the datasets should be directed to the corresponding author. Requests to access these datasets should be directed to Linjun Xie, xielj18@gmail.com.

## References

[1] Scott CS Stark AN Limbert HJ Master PS Head C Roberts BE. Diagnostic and prognostic factors in acute monocytic leukaemia: an analysis of 51 cases. Br J Haematol. (1988) 69:247–52. doi: 10.1111/j.1365-2141.1988.tb07629.x, PMID: 3291931

[2] Ferrando AA Neuberg DS Dodge RK Paietta E Larson RA Wiernik PH. Prognostic importance of TLX1 (HOX11) oncogene expression in adults with T-cell acute lymphoblastic leukaemia. Lancet. (2004) 363:535–6. doi: 10.1016/S0140-6736(04)15542-6, PMID: 14975618

[3] Kees UR Heerema NA Kumar R Watt PM Baker DL La MK. Expression of HOX11 in childhood T-lineage acute lymphoblastic leukaemia can occur in the absence of cytogenetic aberration at 10q24: a study from the Children’s Cancer Group (CCG). Leukemia. (2003) 17:887–93. doi: 10.1038/sj.leu.2402892, PMID: 12750702

[4] Mathews E Laurie T O’Riordan K Nabhan C. Liver involvement with acute myeloid leukemia. Case Rep Gastroenterol. (2008) 2:121–4. doi: 10.1159/000120756, PMID: 21490850 PMC3075178

[5] Gu R Xiang M Jing S Yuan J. Acute lymphoblastic leukemia in an adolescent presenting with acute hepatic failure: A case report. Mol Clin Oncol. (2019) 11:135–8. doi: 10.3892/mco.2019.1877, PMID: 31316772 PMC6604396

[6] Litten JB Rodríguez MM Maniaci V. Acute lymphoblastic leukemia presenting in fulminant hepatic failure. Pediatr Blood Cancer. (2006) 47:842–5. doi: 10.1002/pbc.20544, PMID: 16106432

[7] Heincelman M Karakala N Rockey DC. Acute lymphoblastic leukemia in a young adult presenting as hepatitis and acute kidney injury. J Invest Med High Impact Case Rep. (2016) 4:2324709616665866. doi: 10.1177/232470961666, PMID: 27722178 PMC5036134

[8] Reddi DM Barbas AS Castleberry AW Rege AS Vikraman DS Brennan TV. Liver transplantation in an adolescent with acute liver failure from acute lymphoblastic leukemia. Pediatr Transplant. (2014) 18:E57–63. doi: 10.1111/petr.12221, PMID: 24438382

[9] Anderson SH Richardson P Wendon J Pagliuca A Portmann B. Acute liver failure as the initial manifestation of acute leukaemia. Liver. (2001) 21:287–92. doi: 10.1034/j.1600-0676.2001.021004287.x, PMID: 11454193

[10] Segal I Rassekh SR Bond MC Senger C Schreiber RA. Abnormal liver transaminases and conjugated hyperbilirubinemia at presentation of acute lymphoblastic leukemia. Pediatr Blood Cancer. (2010) 55:434–9. doi: 10.1002/pbc.22549, PMID: 20658613

[11] Spinelli I De Santis A Cesini L Riminucci M Corsi A Forlino M. Acute hepatitis-like presentation with cholestasis of CBFB–MYH11-positive acute myeloid leukemia in an adult male: a case report. J Med Case Rep. (2022) 16:294. doi: 10.1186/s13256-022-03476-7, PMID: 35907896 PMC9339180

[12] Sun K Reynolds RJ Sheu TG Tomsula JA Colton L Rice L. Acute myeloid leukaemia presenting as acute liver failure-a case report and literature review. Ecancermedicalscience. (2019) 13:960. doi: 10.3332/ecancer.2019.960, PMID: 31645888 PMC6786829

[13] Wandroo FA Murray J Mutimer D Hubscher S. Acute myeloid leukaemia presenting as cholestatic hepatitis. J Clin Pathol. (2004) 57:544–5. doi: 10.1136/jcp.2003.013565, PMID: 15113866 PMC1770297

[14] Lee JY Lee WS Jung MK Jeon SW Cho CM Tak WY. Acute myeloid leukemia presenting as obstructive jaundice caused by granulocytic sarcoma. Gut Liver. (2007) 1:182–5. doi: 10.5009/gnl.2007.1.2.182, PMID: 20485638 PMC2871626

[15] Rajeswari B Ninan A Prasannakumari SN Parukuttyamma K. Acute myeloid leukemia presenting as obstructive jaundice. Indian Pediatr. (2012) 49:414–6., PMID: 22700670

[16] Walsh LR Yuan C Boothe JT Conway HE Mindiola-Romero AE Barrett-Campbell OO. Acute myeloid leukemia with hepatic infiltration presenting as obstructive jaundice. Leuk Res Rep. (2021) 15:100251. doi: 10.1016/j.lrr.2021.100251, PMID: 34141563 PMC8184649

[17] Joshi A Kumar R Kulkarni A Mui RK Jha A Hiatt TK. Acute myelomonocytic leukemia: A rare cause of painless jaundice. Cureus. (2025) 17:e77223. doi: 10.7759/cureus.77223, PMID: 39925553 PMC11807341

[18] Maharaj S Chang S. Acute myeloid leukemia presenting with hepatic dysfunction: Should induction be dose reduced? EJHaem. (2024) 5:1092–5. doi: 10.1002/jha2.979, PMID: 39415901 PMC11474342

[19] Bernal W Wendon J. Acute liver failure. N Engl J Med. (2013) 369:2525–34. doi: 10.1056/NEJMra1208937, PMID: 24369077

[20] El-Badrawy A Tawfik A Saleh G Abdel-Naby M Ghazy H Emarah Z. MDCT evaluation of hepatic manifestations in Malignant hematological disorders. Clin Diagn Med. (2020) 2:13–22. doi: 10.31487/j.CDM.2020.02.01

[21] DiNardo CD Jonas BA Pullarkat V Thirman MJ Garcia JS Wei AH. Azacitidine and venetoclax in previously untreated acute myeloid leukemia. N Engl J Med. (2020) 383:617–29. doi: 10.1056/NEJMoa2012971, PMID: 32786187

[22] Pratz KW Jonas BA Pullarkat V Thirman MJ Garcia JS Döhner H. Long-term follow-up of VIALE-A: Venetoclax and azacitidine in chemotherapy-ineligible untreated acute myeloid leukemia. Am J Hematol. (2024) 99:615–24. doi: 10.1002/ajh.27246, PMID: 38343151

[23] Scott LJ. Azacitidine: A review in myelodysplastic syndromes and acute myeloid leukaemia. Drugs. (2016) 76:889–900. doi: 10.1007/s40265-016-0585-0, PMID: 27193945

[24] Salem AH Dave N Marbury T Hu B Miles D Agarwal SK. Pharmacokinetics of the BCL-2 inhibitor venetoclax in subjects with hepatic impairment. Clin Pharmacokinet. (2019) 58:1091–100. doi: 10.1007/s40262-019-00746-4, PMID: 30949874

[25] Van de Louw A Lewis AM Yang Z. Autopsy findings in patients with acute myeloid leukemia and non-Hodgkin lymphoma in the modern era: a focus on lung pathology and acute respiratory failure. Ann Hematol. (2019) 98:119–29. doi: 10.1007/s00277-018-3494-3, PMID: 30218164

[26] Devarapalli UV Sarma MS Mathiyazhagan G. Gut and liver involvement in pediatric hematolymphoid Malignancies. World J Gastrointest Oncol. (2022) 14:587–606. doi: 10.4251/wjgo.v14.i3.587, PMID: 35321282 PMC8919016

[27] Bigildeev AE Shipounova IN Svinareva DA Drize NJ. Leukemia cells invading the liver express liver chemokine receptors and possess characteristics of leukemia stem cells in mice with MPD-like myeloid leukemia. Exp Hematol. (2011) 39:187–94. doi: 10.1016/j.exphem.2010.11.005, PMID: 21094203

[28] Ye H Minhajuddin M Krug A Pei S Chou CH Culp-Hill R. The hepatic microenvironment uniquely protects leukemia cells through induction of growth and survival pathways mediated by LIPG. Cancer Discov. (2021) 11:500–19. doi: 10.1158/2159-8290.CD-20-0318, PMID: 33028621 PMC7858222

[29] Yao S Chai H Tao T Zhang L Yang X Li X. Role of lactate and lactate metabolism in liver diseases (Review). Int J Mol Med. (2024) 54:59. doi: 10.3892/ijmm.2024.5383, PMID: 38785162 PMC11188982

[30] Ramírez-Mejía MM Castillo-Castañeda SM Pal SC Qi X Méndez-Sánchez N. The multifaceted role of bilirubin in liver disease: A literature review. J Clin Transl Hepatol. (2024) 12:939–48. doi: 10.14218/JCTH.2024.00156, PMID: 39544246 PMC11557368

[31] Hawley RG Fong AZ Lu M Hawley TS. The HOX11 homeobox-containing gene of human leukemia immortalizes murine hematopoietic precursors. Oncogene. (1994) 9:1–12., PMID: 7905617

[32] Lichty BD Ackland-Snow J Noble L Kamel-Reid S Dubé ID. Dysregulation of HOX11 by chromosome translocations in T-cell acute lymphoblastic leukemia: a paradigm for homeobox gene involvement in human cancer. Leuk Lymphoma. (1995) 16:209–15. doi: 10.3109/10428199509049759, PMID: 7719228

[33] Durinck K Van Loocke W van der Meulen J Van de Walle I Ongenaert M Rondou P. Characterization of the genome-wide TLX1 binding profile in T-cell acute lymphoblastic leukemia. Leukemia. (2015) 29:2317–27. doi: 10.1038/leu.2015.162, PMID: 26108691

[34] Zweier-Renn LA Hawley TS Burkett S Ramezani A Riz I Adler RL. Hematopoietic immortalizing function of the NKL-subclass homeobox gene TLX1. Genes Chromosomes Cancer. (2010) 49:119–31. doi: 10.1002/gcc.20725, PMID: 19862821 PMC2795049

[35] Perl AE Martinelli G Cortes JE Neubauer A Berman E Paolini S. Gilteritinib or chemotherapy for relapsed or refractory FLT3-mutated AML. N Engl J Med. (2019) 381:1728–40. doi: 10.1056/NEJMoa1902688, PMID: 31665578

[36] DiNardo CD Stein EM de Botton S Roboz GJ Altman JK Mims AS. Durable remissions with ivosidenib in IDH1-mutated relapsed or refractory AML. N Engl J Med. (2018) 378:2386–98. doi: 10.1056/NEJMoa1716984, PMID: 29860938

